# Endovascular Repair of Thoracic Aortic Atresia in Adults: A Narrative Review of a Rare Entity and Emerging Technique

**DOI:** 10.3390/life15111651

**Published:** 2025-10-23

**Authors:** Claudiu Florin Rășinar, Petru Liuba, Alina Diduța Brie, Alexandru Tîrziu, Cristian Mornoș, Daniel Miron Brie, Dan Ion Gaiță, Constantin Tudor Luca

**Affiliations:** 1 Cardiovascular Disease Institute Timisoara, Gheorghe Adam St., No. 13A, 300310 Timisoara, Romania; claudiu.rasinar@umft.ro (C.F.R.); alexandru.tirziu@umft.ro (A.T.); mornos.cristian@umft.ro (C.M.); dan.gaita@umft.ro (D.I.G.);; 2 Doctoral School, “Victor Babes” University of Medicine and Pharmacy Timisoara, Eftimie Murgu Square, 7 No. 2, 300041 Timisoara, Romania; 3 Pediatric Heart Center, Lund University Hospital, Entrégatan 7, 222 42 Lund, Sweden; petru.liuba@pedi.lu.se; 4 Department of Cell and Molecular Biology, “Victor Babes” University of Medicine and Pharmacy, Tudor Vladimirescu Street, No. 14, 300174 Timisoara, Romania; alina.brie@umft.ro; 5 ANAPATMOL Research Center, “Victor Babes” University of Medicine and Pharmacy, Tudor Vladimirescu Street, No. 14, 300174 Timisoara, Romania; 6 Department of Functional Sciences, “Victor Babes” University of Medicine and Pharmacy, Tudor Vladimirescu Street, No. 14, 300174 Timisoara, Romania; 7 Research Center of the Institute of Cardiovascular Diseases, Cardiovascular Disease Institute Timisoara, Gheorghe Adam St., No. 13A, 300310 Timisoara, Romania; 8 Department of Cardiology, “Victor Babes” University of Medicine and Pharmacy Timisoara, Eftimie Murgu Square, No. 2, 300041 Timisoara, Romania

**Keywords:** thoracic aortic atresia, refractory hypertension, adult congenital heart disease

## Abstract

Thoracic aortic atresia in adults represents a rare and extreme manifestation of aortic coarctation, marked by complete luminal occlusion and frequently compensated by extensive collateral circulation. This narrative review critically evaluates existing literature and institutional experience concerning surgical and endovascular repair strategies for aortic atresia, synthesizing evidence from related aortic arch pathologies due to the absence of direct comparative studies. Both treatment modalities—open surgical repair and catheter-based recanalization with stenting—have evolved significantly, presenting distinct advantages and limitations influenced by patient anatomy, age, and comorbidities. While surgical repair remains the standard for neonates, infants, and complex cases due to superior long-term durability and blood pressure control, endovascular procedures using chronic total occlusion technique and covered stents offer less invasive alternatives with rapid recovery, particularly in adults with suitable anatomic characteristics. The review highlights procedural considerations, including technical approaches, stent selection, and potential complications such as restenosis, hypertension, and vascular injury. Individualized, multidisciplinary decision-making remains essential, with shared consensus guiding therapy in the absence of randomized trials. The article identifies critical gaps in knowledge, emphasizing the need for multicenter, long-term studies and technological advances—including hybrid and personalized strategies for optimal management and for improving outcomes in this challenging congenital condition.

## 1. Introduction

Coarctation of the aorta involves narrowing of the thoracic aorta at the isthmic level, the site of insertion of the fetal ductus arteriosus, and it is one of the most common congenital malformations (0.04% in the general population, 10% of adult congenital heart defects), with a higher prevalence in males [1,2,3,4,5]. In most cases it is associated with other congenital malformations, the most common association being bicuspid aortic valve (85%), mitral valve malformations, or ventricular septal defects [5]. Signs and symptoms depend on the severity of the coarctation, the most common manifestation being arterial hypertension in the upper limbs.

Aortic atresia is a rare and extreme form of aortic coarctation in which the connection between the aortic arch and the descending aorta is made through an atretic, fibrous segment. The differential diagnosis must be made with the interrupted aortic arch, in which this fibrous, atretic connection is missing. This is important because few patients with aortic arch interruption survive up to a year (mortality at one year 72%), and the treatment is solely surgical [6,7].

Although in the past the treatment of aortic atresia was exclusively surgical, most cases can now be addressed using endovascular approaches, often employing interventional techniques derived from the treatment of chronic coronary occlusions. Both surgical and endovascular approaches have evolved as treatment options, each with distinct advantages and limitations.

The choice between surgical and endovascular intervention depends on numerous factors including patient anatomy, age, previous interventions, and institutional expertise. Understanding the comparative outcomes, complications, and long-term durability of these approaches is essential for optimal patient management and treatment planning.

### 1.1. Purpose and Scope of the Review

The objective of this report is to critically evaluate the existent research on “comparing surgery versus catheter-based techniques for treating aortic atresia” to synthesize current evidence regarding the safety, efficacy, and long-term outcomes of these treatment modalities, as aortic atresia presents significant clinical challenges, and optimal management strategies remain contested due to the evolution of surgical and catheter-based techniques. By systematically analyzing comparative studies, including hybrid and minimally invasive approaches, this paper aims to elucidate the relative benefits and limitations of each technique. Ultimately, this synthesis will inform clinical decision-making, identify gaps in knowledge, and guide future research directions to enhance patient outcomes in this complex congenital condition.

Specific Objectives:

1. To evaluate current knowledge on the safety profiles and procedural success rates of surgical versus catheter-based treatments for aortic atresia.

2. To benchmark existing approaches to assess long-term morbidity, mortality, and reintervention rates following different aortic atresia interventions.

3. To identify and synthesize clinical outcome measures used in studies comparing hybrid, minimally invasive, and conventional surgical techniques.

4. To compare perioperative and postoperative complications associated with catheter-based and surgical interventions in aortic atresia patients.

5. To deconstruct patient selection criteria and risk stratification methods influencing treatment modality choice in aortic atresia management.

### 1.2. Methodology of Literature Selection

A literature review across multiple databases, including PubMed, EMBASE, and Google Scholar, with a focus on comparative studies, outcomes data, and long-term follow-up reports was performed.

The initial research query—“comparing surgery versus catheter-based techniques for treating aortic atresia”—was transformed into a series of more specific search statements. The following queries derived from the original inquiry: “Comparing surgery versus catheter-based techniques for treating aortic atresia”; “Evaluating minimally invasive and hybrid techniques for aortic interventions: outcomes and effectiveness in comparison to surgical approaches”; “Evaluating hybrid and minimally invasive techniques for aortic interventions: outcomes and comparisons in aortic atresia treatments”; “Assessing the efficacy and outcomes of hybrid and minimally invasive interventions in aortic atresia treatment compared to conventional surgical techniques”; “Examining hybrid surgical and catheter-based approaches for aortic atresia: a comprehensive comparison of clinical outcomes and effectiveness”.

This literature review identified a critical evidence gap: no published studies directly compare surgical versus endovascular treatments for aortic atresia, highlighting the urgent need for collaborative research in this rare but life-threatening condition.

PICO Question

Population (P): Patients of all ages diagnosed with aortic atresia (extreme form of aortic coarctation)

Intervention (I): Surgical repair (including resection with end-to-end anastomosis, patch repair, bypass grafting)

Comparison (C): Endovascular intervention (balloon angioplasty, covered stent placement, bare metal stent placement)

Outcome (O): Primary outcomes: procedural success, mortality, re-intervention rates; Secondary outcomes: gradient reduction, complications, long-term patency, quality of life, functional status

Study Design: Randomized controlled trials, comparative cohort studies, case–control studies, prospective and retrospective comparative studies

Time Frame: Studies published between 2000–2025

Research Question: What are the comparative effectiveness, safety, and long-term outcomes of surgical repair versus endovascular stent placement for treatment of aortic coarctation?

Inclusion Criteria

1. Studies including patients with confirmed diagnosis of aortic coarctation (native or recurrent)
2. Studies directly comparing surgical repair versus endovascular intervention (stent placement or balloon angioplasty) OR studies reporting outcomes for either treatment modality that allow for indirect comparison
3. Original research articles including randomized controlled trials, cohort studies, case–control studies, and prospective/retrospective observational studies
4. Studies published in English between January 2000 and July 2025
5. Studies with clear treatment outcome measures (mortality, morbidity, recurrence, re-intervention rates)
6. Studies with minimum sample size of 10 patients
7. Human studies only
8. Studies reporting follow-up data of at least 30 days post-intervention
9. Studies with clearly defined patient populations and treatment protocols

Exclusion Criteria

1. Animal studies, in vitro studies, or experimental models
2. Case reports or case series with fewer than 10 patients
3. Editorials, opinion pieces, narrative reviews, and commentaries
4. Conference abstracts without full-text availability
5. Duplicate publications or studies with overlapping patient datasets
6. Studies focusing primarily on other congenital cardiac defects without specific focus on aortic coarctation or atresia
7. Studies without clinical outcomes or treatment-related endpoints
8. Studies published before January 2000
9. Studies not published in English
10. Studies with insufficient data for outcome assessment
11. Reviews and meta-analyses

We have screened 708 papers across all databases. The title and abstract screening of the supplied records found zero studies meeting the predefined inclusion criteria and all screened records were excluded. The supplied set therefore contains no directly includable primary or comparative studies of surgical versus endovascular treatment for aortic atresia.

The primary reasons for exclusion were as follows: incorrect target condition (44.1%—standard coarctation versus atresia), lack of a comparative design (26.3%—single-arm studies), inappropriate study population (13.8%—adults only, other congenital heart diseases), unsuitable study design (9.5%—case reports, reviews), and language or publication restrictions (6.4%).

No direct comparative evidence between surgical and endovascular approaches for aortic atresia was identified among the screened abstracts, so there are no pooled or directly comparative results to report for that specific condition. Although no studies were included, several high-quality comparative or meta-analytic studies from related aortic conditions were identified during the screening process and are the best candidates to inform background, comparative methods, and expected outcome domains if aortic atresia-specific studies become available.

### 1.3. Anatomy and Embryology

Aortic arch atresia is a rare congenital cardiac anomaly characterized by a segment of the aortic arch that is anatomically present but completely occluded, lacking luminal patency and often replaced by a fibrous strand [8]. The precise etiology remains unclear [9], but current understanding is based on two main theories and clinical observations.

1. Embryological and Developmental Theories

Skodiac theory (ductus tissue theory)

In ductus tissue theory, it is believed that the closure of ductus arteriosus extends into the aortic wall. In a normal case, without coarctation, the junction between the ductus arteriosus and the aortic wall is minimal. However, in case of coarctation/atresia patients, a significant amount of ductal tissue is infiltrated in the aortic wall in the form of overlapped smooth muscle rings that surround the aortic wall. Supporting evidence comes from case reports describing neonates with CoA who received prostaglandin E1 after ductus arteriosus closure, with improved hemodynamic parameters and without ductus arteriosus reopening [10,11]. Other supporting studies based on histopathological data suggest that in coarctation segments, ductal tissue (of the internal elastic lamina disassembly and fragmentation and sparse elastic fibers in the middle layer) borders more than half of the aortic circumference, compared to less than one third of the aortic circumference in normal patients [12]. Furthermore, Kim et al. found terminal dUTP nick end labeling (TUNEL)-positive cell deaths in the intima and media of both coarctation segments and the ductus arteriosus but none in normal aortic tissue [13].

Disordered embryogenesis of the aortic arch

Congenital aortic arch anomalies, including atresia, are believed to result from the abnormal persistence or involution of embryonic vascular segments during development. This can lead to either complete absence (interruption) or fibrous atresia of a segment of the arch [14]. Late developmental changes: atresia often occurs at the distal end of the left subclavian artery, suggesting that it arises after the seventh intersegmental artery has migrated. This indicates that the isthmus was initially normal but later became occluded [9,15].

Smooth Muscle Cell Migration Theory

One hypothesis suggests that smooth muscle cells from the ductus arteriosus migrate into the aortic wall during embryogenesis. After birth, closure of the ductus arteriosus leads to contraction and fibrosis, potentially causing stenosis or atresia at the isthmus. Ductal smooth muscle cells present a faster differentiation to a mature smooth muscle cell phenotype, required for contraction and immediate closure after birth. In the media of coarctation segments, the smooth muscle phenotype is similar to that of ductus arteriosus [12].

Hemodynamic theory

Another theory suggests that reduced blood flow through the aortic isthmus during fetal development leads to aortic underdevelopment (hypoplasia) and subsequent atresia. Increased stiffness, excessive collagen production, and cystic media necrosis in the vessel wall of affected neonates support this affirmation [16,17].

2. Acquired and Secondary Factors: Inflammation and maternal factors

There is evidence that postnatal inflammatory processes can exacerbate pre-existing coarctation or hypoplasia, potentially leading to complete atresia. Maternal conditions such as gestational diabetes mellitus may increase the risk of aortic arch atresia in the newborn. Histopathological findings from surgical and pathological examinations often reveal mucoid degeneration, poor muscular structure, and granulation tissue in the atretic segment, indicating both congenital developmental defects and secondary degenerative changes [18]. Comparative transcriptomic analysis between healthy (proximal and distal tissue) and coarctation aortic segment biopsies describe an upregulation of neuropilin-1 (NRP-1, VEGF receptor), complement components (C1, C3), and cytokines involved in acute phase responses (IL-6, IL-23), and inflammatory factors like IκBζ, CEBPD, ATF3, and DUSP1 in the coarctation segments. Furthermore, it was found that patient characteristics like sex and age significantly influence the transcriptome of the coarctation area. Males show a response more inclined towards interferon, while females exhibit a response more inclined towards inflammation. S100 signaling, particularly S100A8/A9 and S100A12, was found to be elevated in males, leading to IRF activation and interferon production, while females showed higher expression of S100A1 and S100A7, driving increased inflammation. This response variance is potentially influenced by estrogen and NO signalling [19].

3. Associated Cardiac Anomalies

Aortic arch atresia is frequently found in conjunction with other congenital heart defects, such as persistent ductus arteriosus, ventricular septal defect, and aortic valve abnormalities [9].

Aortic arch atresia in adults is extremely rare and often presents with subtle or nonspecific symptoms due to the development of extensive collateral circulation over time, which compensates for the obstructed segment.

## 2. How to Approach a Patient with Thoracic Aortic Atresia

### 2.1. Diagnosis

#### Clinical Manifestations

A significant number of adults remain asymptomatic due to the presence of collateral vessels that circumvent the atretic segment, thereby preserving distal perfusion.

However, this condition is often diagnosed during the assessment of hypertension that is difficult to manage. Blood pressure measurements in all four limbs should be performed, as there is often a pronounced difference in blood pressure recordings between the upper and lower extremities, with significantly higher measurements in the arms compared to the legs. This clinical finding must be confirmed by invasive blood pressure measurement. Some patients may experience leg fatigue or pain (intermittent claudication) during physical exertion, which is usually attributed to diminished blood flow to the lower extremities. Upon examination, femoral and other lower limb pulses may be weak or non-palpable, whereas upper limb pulses remain normal. In more advanced stages, symptoms such as exertional dyspnea, orthopnea, and peripheral edema may manifest, indicative of congestive heart failure resulting from prolonged pressure overload. Non-specific symptoms, including headache and general malaise, may also be observed and may be attributed to uncontrolled blood pressure values. As the presence of an atretic segment increases the afterload, myocardial oxygen consumption increases and simultaneously, pathological left ventricular hypertrophy occurs, additionally altering the balance between oxygen supply and demand. That is why myocardial ischemia may occur, particularly in the presence of concomitant coronary artery disease. If left untreated, aortic atresia can result in severe sequelae such as stroke, aortic dissection, coronary artery disease, and premature death, akin to severe coarctation of the aorta [20].

**Figure 1:**
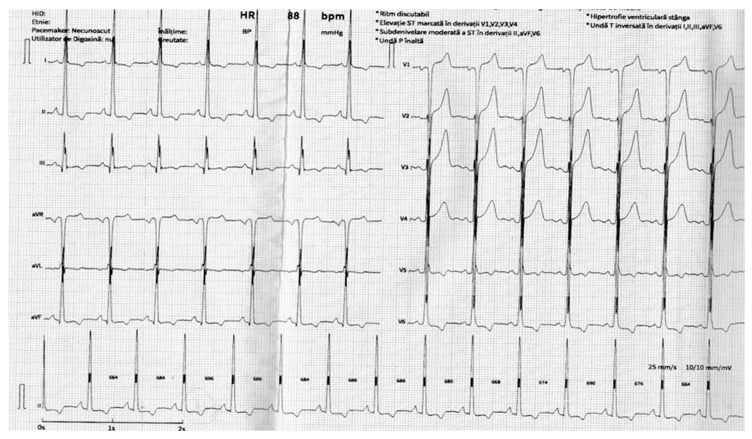
The 12-lead electrocardiogram (ECG) of a patient diagnosed with aortic atresia—sinus rhythm, a heart rate of 88 bpm, and suggestive signs of left ventricular hypertrophy (LVH)—strain pattern in I, II, aVL, aVF, V5, V6.

The electrocardiographic aspect (Figure 1) may be influenced by the patient’s age, anatomy and comorbidities, and may range from signs of right ventricular hypertrophy (right QRS axis deviation, with a prominent R wave in leads V1–V3, deep S wave in leads V5-V6, R/S ratio > 1) and (in)complete right bundle branch block in the neonates and younger patients to left ventricular hypertrophy (with ST-T wave changes) and left atrial abnormalities (including atrial fibrillation) in older patients.

**Figure 2:**
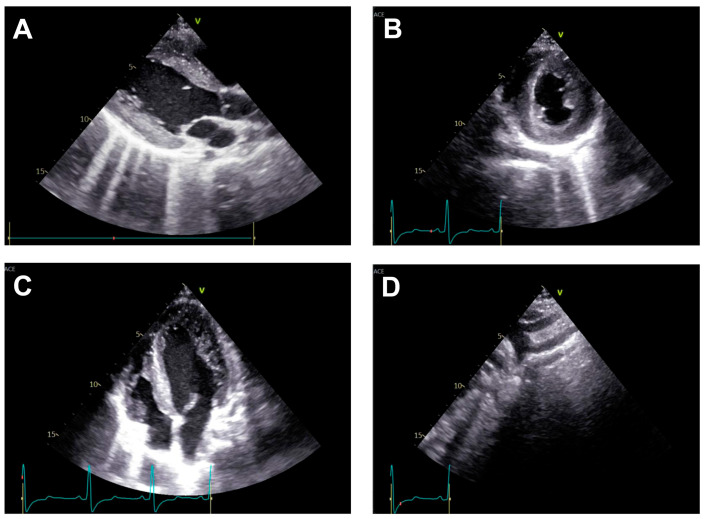
Transthoracic echocardiographic aspect. (**A**). Parasternal long-axis view at the level of the left ventricle (LV)—significant left ventricular hypertrophy (LVH) with normal morphology of the aortic valve (AV) and mitral valve (MV). (**B**). Parasternal short-axis view at the level of the papillary muscles, confirming left ventricular hypertrophy (LVH) without evidence of pericardial effusion. (**C**). Apical four-chamber view further highlighting pronounced left ventricular hypertrophy (LVH). (**D**). Suprasternal window showing the emergence of the left common carotid artery and left subclavian artery, followed by abrupt narrowing and total occlusion of the descending thoracic aorta.

Transthoracic echocardiography (Figure 2) may reveal signs of left ventricular hypertrophy and diastolic dysfunction from the apical and parasternal views, while the suprasternal view may identify the aortic atretic segment. However, the presence of collateral circulation is difficult to identify using this technique [21].

**Figure 3:**
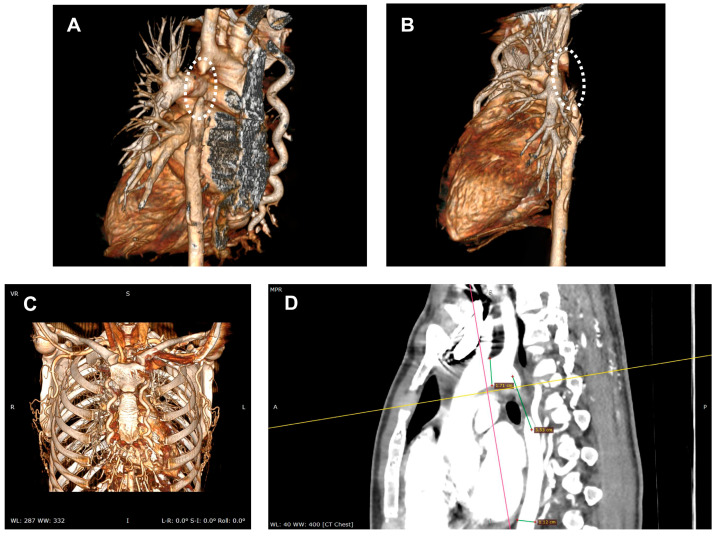
(**A**,**B**). Computed tomography angiography with three-dimensional reconstruction identified an atretic segment in the descending thoracic aorta (dashed circle). (**C**). Computed tomography angiography with three-dimensional reconstruction demonstrating a well-developed collateral circulation between the proximal and distal portions of the atretic aortic segment; (**D**). Sagittal view showing a proximal aortic diameter of 1.71 cm, a descending aortic diameter of 1.12 cm, an atretic segment measuring approximately 2.5 cm in length, and a stent landing zone of 3.53 cm.

Diagnosis is often incidental, made during workup for hypertension or vascular complaints, and confirmed by imaging (CT—Figure 3, MRI, echocardiography) showing the atretic aortic segment and collateral vessels.

### 2.2. Treatment

Aortic arch atresia (AAA) is a rare congenital defect requiring individualized treatment strategies based on patient age, anatomy, and comorbidities. Mean untreated survival is approximately 35 years with most deaths by the fifth decade. The most frequent cardiovascular endpoints are uncontrolled hypertension, left-ventricular hypertrophy and failure, aortic aneurysm/pseudoaneurysm with risk of rupture or dissection, and premature coronary atherosclerosis [22].

Management options include both surgical repair and percutaneous (catheter-based) interventions.

In the absence of comparative evidence, treatment decisions for aortic atresia currently rely on:

1. Individual clinical expertise and institutional experience
2. Extrapolation from related conditions (standard coarctation)
3. Single-center case series with inherent selection bias
4. Theoretical considerations based on anatomic principles

Surgical treatment of aortic arch atresia is indicated in specific clinical scenarios, primarily determined by the patient’s symptoms, anatomy, age, and associated cardiovascular conditions.

Interventional repair of aortic arch atresia is indicated in symptomatic patients with significant obstruction or complications, with the choice of technique tailored to patients’ anatomy, age, and comorbidities. Percutaneous approaches are increasingly feasible in adults, while surgery remains the primary treatment option in infants and complex cases.

Surgery is indicated in infants with aortic arch atresia who are symptomatic, ductal-dependent, have significant associated cardiac anomalies, or cannot be managed with catheter-based approaches [23]. Early diagnosis and prompt surgical intervention are essential for survival and optimal outcomes in infants. In adults, surgical procedures are typically reserved for symptomatic individuals or those experiencing complications, particularly when percutaneous options are not viable [20]. The presence of concomitant cardiac anomalies often necessitates a surgical approach to facilitate comprehensive correction. Surgical interventions should be conducted in specialized centers due to the inherent complexity and associated risks. The surgical repair of aortic arch atresia may involve extra-anatomic bypass, direct patch augmentation, or intricate procedures, with the choice determined by patient age, anatomical considerations, and associated cardiac lesions. Single-stage extra-anatomic ascending-to-descending aortic bypass graft surgery is performed particularly in adults with symptomatic heart failure and related cardiac conditions. Direct repair or reconstruction of the atretic segment in infants, especially when the atresia is located distal to the left subclavian artery (type I), restores aortic continuity [24].

Percutaneous recanalization and stenting have emerged as viable, safe, and effective alternatives to surgery for selected patients with aortic arch atresia, particularly adults with suitable anatomical features. In the most significant available multicenter cohort (19 adult patients, median follow-up 4.94 years), percutaneous recanalization with covered stent implantation resulted in good long-term survival. Only one patient died during follow-up, and this was believed to be unrelated to the intervention [20]. Lifelong follow-up is essential due to the risk of restenosis and the need for potential reintervention, as well as to monitor blood pressure and cardiac function. Long-term follow-up after percutaneous recanalization of aortic arch atresia demonstrates good survival, improved blood pressure control, and acceptable rates of restenosis requiring reintervention. Lifelong, structured follow-up is essential to ensure optimal outcomes and the timely management of complications [20]. Hypertension resolution after recanalization of aortic arch atresia depends on a combination of patient age, arch anatomy, cardiovascular risk profile, and medical management. Early intervention and comprehensive post-procedural care improve the likelihood of normalizing blood pressure. Younger patients have a higher likelihood of hypertension resolution post-recanalization. Older age is associated with persistent hypertension due to vascular remodeling and loss of compliance.

Endovascular techniques for AAA present a minimally invasive alternative to open surgery, particularly advantageous for adults or high-risk patients with appropriate anatomical conditions. These methods aim to recanalize the atretic segment and restore aortic continuity while maintaining flow to the supra-aortic branches.

While no direct comparative studies exist for aortic atresia, related literature on standard aortic coarctation show similar short-term technical success rates between approaches, lower procedural morbidity with endovascular techniques, higher reintervention rates with balloon dilation vs. surgery, and improved outcomes with covered stent technology [25].

#### 2.2.1. Core Steps of Endovascular Technique for AAA

For endovascular treatment of AAA, patients usually undergo the procedure under general anesthesia with orotracheal intubation [20,26]. The cardiac surgery team and heart-lung machine remain on standby in the hybrid room throughout the intervention.

Two arterial accesses are required for this intervention: one above the atretic segment—from the upper limbs (radial, ulnar or brachial access), and one below the atretic segment—femoral access. In case of radial artery, severe arterial spasm may occur, requiring a more proximal access—the brachial artery.

Simultaneous contrast injection via an MPA2 6F catheter at the proximal descending thoracic aorta and a retrograde injection via a Pigtail 6F catheter at the diaphragmatic level reveal a narrowing of the aortic lumen, followed by total occlusion.

#### 2.2.2. Crossing the Atretic Segment

The most important part of the procedure is crossing the atretic segment and creating a connection between the two ends of the aorta. Crossing the atretic segment can be performed with a radiofrequency (RF) system—a Nykanen 0.024″ RF guidewire (Baylis Medical, Montreal, QC, Canada) advanced through a coaxial catheter (multipurpose or right Judkins coronary artery catheter) to perforate the atretic segment by delivering controlled bursts of energy (up to 20 W for 3–5 s). Another approach is to use extra-stiff guidewires, such as Cross-IT 300 (Guidant, Indianapolis, IN, USA), ACS BMW 0.014″ (Abbott Vascular, Chicago, IL, USA), GLIDEWIRE^®^ Hydrophilic Coated Guidewire (Terumo, Somerset, NJ, USA), or Platinum Plus™ 0.018″ (Boston Scientific, Marlborough, MA, USA) to mechanically perforate the atretic tissue by pushing the stiff tip through the occlusion.

In the case of pediatric patients or occluded coarctations, a pediatric Brockenbrough needle (Medtronic) can be used to carefully puncture the caudal atretic end under fluoroscopic guidance. Consequently, an arterio-arterial circuit is established between femoral and brachial/radial access points to facilitate device delivery.

RF systems (Nykanen/Baylis) are designed for controlled tissue perforation with published adult atresia series showing 100% technical success, large gradient reduction, and no immediate complications in a small cohort. In this report, a Nykanen 0.024″ RF perforation wire advanced inside a coaxial microcatheter was used to perforate the atretic isthmus; after successful crossing, the wire was snared to create an arterio-arterial rail, predilatation performed with coronary balloons, then a long sheath advanced for covered stent implantation. Percutaneous recanalization using the Nykanen RF system succeeded in all subjects, with a mean fluoroscopy time reported to 30 ± 6 min and mean procedure time 90 ± 15 min. In terms of hemodynamic and clinical results, pre-stent transcoarctation gradient fell from a mean of ~52 mm Hg to ~3 mm Hg after covered stent implantation, and systemic blood pressure improved with reduced antihypertensive requirements during a mean follow-up of 19 months. No immediate complications were reported in the series using the Nykanen RF wire and covered CP stents, and no significant complications during mid-term follow-up were documented in the same cohort [27].

In the case of Brockenbrough needle technique, a pediatric transseptal puncture needle is used to permeabilize totally occluded coarctations, followed by covered stent implantation in the same session. In a small paediatric series of three children (ages 9–14 years) with complete aortic occlusion, recanalization and covered stent implantation after crossing with a Brockenbrough needle were successful in all three patients; one patient developed transient post-coarctectomy syndrome. Brockenbrough needles are considered mechanically effective in small pediatric series and feasible for immediate stent delivery, where an antegrade mechanical puncture is considered safe. However, post-recanalization syndromes must be closely monitored [28].

Another option are the high-support coronary wires (for example Conquest Pro 12) to perforate atretic vessels after RF failure. However, the evidence for aortic atresia is limited to small reports [29]. High-support wires rely on mechanical penetration and carry theoretical risk of uncontrolled perforation or injury to adjacent structures. Reported clinical data show feasibility but are numerically small and include one transient complication with Brockenbrough use in children [28]. Extra-stiff coronary wires can penetrate resistant atretic tissue when RF fails. It must be used by experienced operators with awareness of perforation risk and lack of large aortic atresia studies [29].

Antegrade wire escalation, similar to that used in CTO procedures, is attempted using coronary Pilot 50 or Pilot 150 guidewires (Abott Vascular). In case of difficult atretic segment crossing, an Asahi Confianza Pro 12 coronary guidewire (Asahi Intecc) can be considered.

Contrast injection from the right femoral artery pigtail catheter confirms the positioning in the true aortic lumen.

The Confianza Pro guide (Asahi Intec) is then replaced with a BMW (Abbott Vascular) 300 cm guide using a 4F FineCross microcatheter (Terumo). The BMW 0.014″ 300 cm guidewire is then advanced through the atretic portion in the abdominal aorta. The distal tip of the guidewire is captured using a snare device (Amplatz Goose Neck, Medtronic) and externalized via right femoral access, creating an aorta-to-aorta connection.

#### 2.2.3. Balloon Angioplasty

Following successful crossing, the atretic segment is sequentially dilated using coronary or peripheral angioplasty balloons to create a channel for stent placement.

#### 2.2.4. Stent Placement

A covered stent, sized according to the diameter of the distal aorta and arch, is deployed across the recanalized segment to restore antegrade flow. Balloon inflation ensures full expansion and apposition of the stent. Over the BMW guidewire a Multipurpose A2 catheter is retrogradely advanced proximally to the coarctation. The BMW guidewire is then replaced with a SuperStiff guidewire (Amplatzer, Boston Scientific), positioned within the ascending aorta. Following this, an introducer sheath system (D’Vill 12F, NuMed Inc., New York, NY, USA) is advanced, with the guidewire and dilator subsequently removed.

A Pigtail 6F catheter used for antegrade contrast injection via the proximal access (radial/brachial) confirms the correct placement of the proximal end of the introducer sheath.

Finally, a 45 mm Cheatham Platinum covered stent (NuMed Inc., New York, NY, USA) is mounted on a BIB 12/45 mm balloon (NuMed Inc., New York, NY, USA) and deployed at the coarctation site.

#### 2.2.5. Branch Vessel Management

If the atresia involves or is proximal to the supra-aortic branches, techniques such as chimney, fenestrated, or in situ fenestration may be employed to maintain perfusion to the head and neck vessels. In situ fenestration involves puncturing the stent graft from within the vessel to create a channel for branch vessel stenting.

#### 2.2.6. Imaging and Hemodynamic Assessment

Final angiography confirms the stent position, patency of the aorta and branch vessels, and the absence of leaks or dissections.

**Figure 4:**
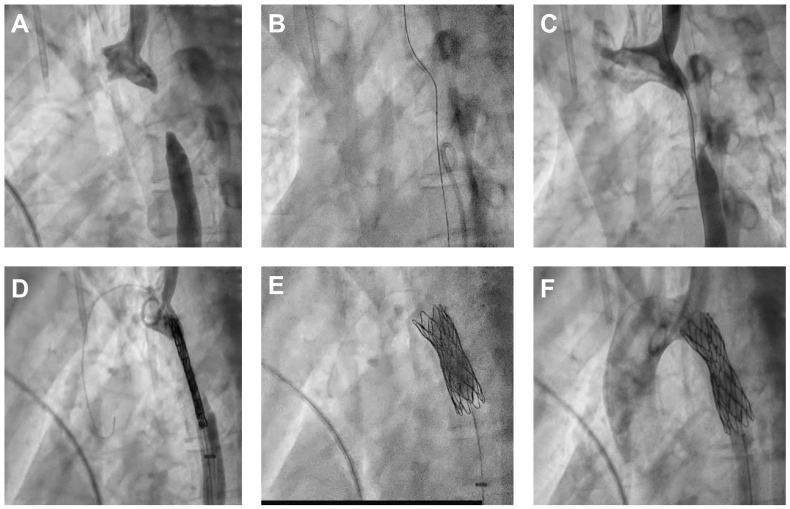
(**A**). Antegrade injection through an MPA2 6F catheter at the level of the aortic arch, and retrograde injection through a Pigtail 6F catheter at the level of the descending thoracic aorta, distal to the atretic segment. No patent lumen is observed within the atretic segment, which extends over approximately 35 mm. (**B**). A JR 6F guiding catheter is positioned at the level of the atretic segment. The Confianza Pro guidewire is gradually advanced through the atretic zone, extending distally beyond it. (**C**). Injection at the aortic level confirms the recanalization of the atretic segment. The BMW 300 guidewire, introduced via the left brachial approach through the left subclavian artery, successfully crosses the coarctated segment and is externalized at the right femoral access site. (**D**). The stent is positioned at the level of the coarctated segment. The D’Vill introducer is retracted distal to the coarctation site. A contrast injection via the Pigtail catheter is performed to confirm the correct positioning of the stent’s proximal end relative to the left subclavian artery. (**E**). A waist persistence is observed in the mid-section of the stent, indicating residual narrowing. A post-dilatation is performed using a 12/20 mm balloon at nominal pressure, achieving adequate expansion of the stent. (**F**). Injection via the Pigtail catheter demonstrates persistent blood flow through the stented area, with no evidence of dissection proximally or distally to the stented segment.

Following stent deployment, blood pressure measurements proximal and distal to the stent are ideally equal, with no residual pressure gradient. Angiographic assessment of the aortic arch demonstrated a well-expanded and well-positioned stent must be with no signs of proximal or distal dissection (Figure 4).

### 2.3. Post-Procedure Care

Patients are monitored for 48–72 h, receive prophylactic antibiotics, and are typically prescribed antiplatelet therapy (e.g., aspirin) for several months.

There are no standardized protocols for aortic atresia in the current literature and data must be extrapolated from single case reports or case series. Endovascular case reports focus on two phases of antithrombotic care: an intra-procedural anticoagulation and post-procedural antiplatelet/anticoagulant therapy, akin to catheterization lab antithrombotic therapy norms used for stent procedures (intravenous heparin during procedure) and post-procedure antiplatelet therapy according to local practice and patient factors, weighing the balance between thrombotic and bleeding risks [30]. An intravenous bolus of 5000 IU unfractionated heparin is administered during stent deployment, followed by single antiplatelet therapy with aspirin 100 mg daily after stent implantation [31] or dual anti-platelet therapy (aspirin plus clopidogrel) for up to 6 months [32].

**Figure 5:**
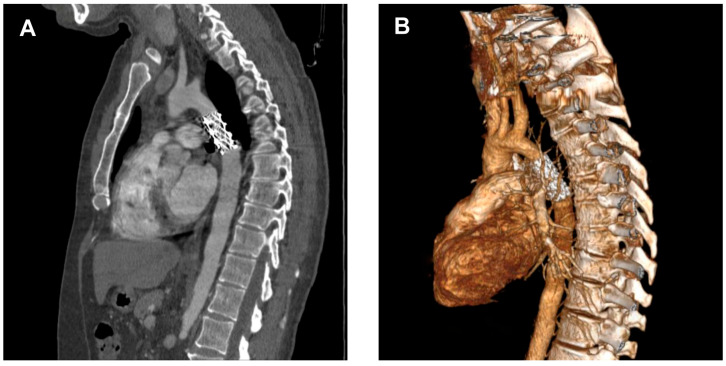
Six-month follow-up imaging after endovascular treatment of thoracic aortic atresia. (**A**) Sagittal reconstruction of contrast-enhanced computed tomography (CT) demonstrates patency of the thoracic aortic stent-graft with preserved alignment and no evidence of endoleak. (**B**) A three-dimensional volume-rendered CT image confirms the proper stent position and unobstructed aortic flow, with no signs of graft migration or stenosis.

Angio CT (CT angiography) is typically performed at specific intervals after aortic coarctation repair with stent implantation to assess stent position, vessel patency, and rule out complications such as restenosis, aneurysm, or stent migration. Angio CT is usually recommended within the first 1–3 months after stent placement to verify correct stent expansion, position, and absence of immediate complications. After the initial scan, repeat angio CT or MRI is generally performed at 6–12 months after intervention to monitor for restenosis, vessel remodeling, or aneurysm formation. The choice between angio CT, MRI (preferred in younger patients due to lack of radiation), or echocardiography depends on age, stent type (metal artifacts), and clinical scenario [33] (Figure 5).

#### 2.3.1. Surgery vs. Endovascular Treatment

The main indication for surgery in aortic atresia and aortic coarctation is the presence of complex anatomy, severe or long-segment obstruction, associated cardiac or vascular anomalies, or failed/unsuitable endovascular approach. Surgical repair is favored in neonates, young children, or cases with hypoplastic aortic arch, long-segment atresia, or complex aortic lesions. In contrast, endovascular treatment is indicated for discrete, short-segment coarctation in older children, adolescents, and adults with favorable anatomy and vessel size, offering shorter recovery times and effective relief of luminal narrowing [34].

Surgery is preferred when the vessel size is too small for stent delivery, anatomy precludes safe or complete coverage with endovascular devices, there is concomitant cardiac pathology (e.g., ventricular septal defect, complex congenital heart disease), or when prior stenting or balloon dilation has failed or produced complications such as aneurysm, rupture, or restenosis [34,35].

Surgery is preferred for aortic atresia when anatomical features include extensive or long-segment atresia, complete occlusion with no luminal continuity, severe hypoplasia of the aortic arch, involvement of critical collateral circulation, associated complex congenital cardiac anomalies (such as large ventricular septal defect, mitral apparatus hypoplasia, or hypoplastic left ventricle), or when endovascular access is technically not feasible due to vessel size, tortuosity, or prior complications. Surgical repair is also indicated when the anatomy poses a high risk for rupture or inadequate stent deployment, or when significant bleeding risk due to collaterals makes endovascular approaches hazardous [20,36].

These features typically require open surgical techniques for direct reconstruction, extra-anatomic bypass, or patch plasty, especially in neonates and infants where vessel diameters are small and long-term durability is of utmost importance. In contrast, adults with shorter segment acquired atresia and adequate collateral flow may benefit from endovascular options, provided anatomical access and suitability allow for safe intervention.

Endovascular stent placement is typically considered for native or recurrent coarctation when anatomy is accessible, patient size permits device delivery, and risks of wall injury or adjacent branch vessel occlusion are low, with best outcomes seen in patients over 30 kg [20,37].

Endovascular treatment methods for aortic atresia primarily include CTO-style wire recanalization, balloon angioplasty, and stent (especially covered stent) implantation—these options are considered when the atretic segment is short, localized, and accessible, and when patient anatomy allows safe catheter access and device deployment, typically in older children or adults with adequate vessel size and collateral circulation [38]. Recanalization often begins with specialized guidewire or electrified wire techniques to cross the atresia, followed by controlled balloon angioplasty to dilate the segment, then covered stent placement to restore vessel continuity and prevent acute complications such as rupture or aneurysm formation—these methods are preferred when the anatomy is favorable, surgical risks are high, or previous surgical/interventional therapies have failed. Each method should be tailored to individual patient characteristics, with careful imaging and multidisciplinary planning essential to maximize procedural success and long-term outcomes [20].

Radiofrequency wire perforation and electrified guidewire techniques are currently the most effective endovascular recanalization methods for complete aortic isthmus atresia—these approaches employ specialized radiofrequency guidewires (such as the Nykanen or PowerWire systems) or a denuded guidewire connected to electrosurgical energy to safely and precisely cross the atretic segment, allowing for subsequent balloon angioplasty and covered stent implantation. This technology is particularly valuable when standard guidewires cannot traverse the occlusion and is now used successfully in both adult and pediatric cases [38].

Chronic total occlusion (CTO) coronary methods—including specialized guidewires, microcatheters, antegrade and retrograde approaches, and wire escalation techniques—can be adapted to the endovascular treatment of aortic atresia by facilitating safe and effective recanalization of completely occluded aortic segments, particularly when traditional wire passage is unsuccessful or vessel anatomy is complex. Dedicated CTO guidewires with high penetration power, steerability, and torque control allow precise navigation and controlled perforation through atretic or fibrotic tissue, supported by microcatheters that enhance wire support and coaxial alignment for accessing the occluded segment [39].

Operators frequently use wire escalation, starting with softer wires for initial probing, then progressing to stiffer wires for crossing the atresia segment, similar to complex coronary CTO recanalization strategies; orthogonal imaging and careful manipulation are essential for minimizing risks like vessel perforation. Retrograde techniques, pioneered in coronary CTO practice, may also be applied if collateral or alternative access channels are available, and the technology is particularly valuable when completing percutaneous recanalization as a bridge to balloon angioplasty and stent placement [40].

Favorable anatomical features predicting better outcomes with transcatheter stenting after aortic arch or isthmus recanalization include a short-segment or focal lesion, minimal vessel tortuosity, adequate proximal and distal landing zones (at least 1 to 1.5 cm of disease-free aorta [41]), large enough vessel diameter to accommodate the stent, absence of severe hypoplasia or major branching at the lesion site, and robust peripheral vasculature that allows safe device placement. Native coarctation and atresia situated away from the origins of critical branch vessels, as well as lesions with low collateral burden, have demonstrated higher procedural success and reduced risk for complications, restenosis, or stent migration [20,42].

Use of covered stents is particularly advantageous in cases with fragile vessel walls, aneurysm association, or genetic aortopathies, providing an extra protective barrier against rupture. In summary, outcomes are best when anatomy presents with discrete, short occlusions, allows for optimal stent sizing, and does not compromise essential vascular branches or future vessel growth [43].

A comparative analysis between the surgical and endovascular repair for aortic atresia is summarized in Table 1.

**Table 1:** Comparative analysis between surgical and endovascular repair for aortic atresia.

Parameter	Surgical Repair	Endovascular Treatment
Procedural Success Rate	98.7% (1-year freedom from reintervention) [34]	97% (pooled technical success) [44]
Early Mortality (30-day)	3.9–8.6% (varies by complexity) [45]	2.7–3.9% (generally lower) [45]
Late Mortality (5-year)	Variable by indication	98.1% survival at 5 years [46]
Major Neurological Complications	Higher stroke risk in complex cases [47]	Reduced paraplegia (RR 0.70)
Hospital Stay	7–14 days (longer ICU stay) [48]	2–5 days (shorter recovery) [48]
Restenosis/Recoarctation	5–15% at 10 years [49]	10–25% at 5–10 years
Reintervention Rates	2–8% at 5 years [50]	15–30% at 10 years [50,51]
Blood Pressure Control	Excellent long-term (85–90%) [52]	Good short-term (80–85%)
Procedural Mortality	1–3% (isolated CoA) [53]	0.5–2% (balloon/stent)
Cost-Effectiveness	Higher initial cost, lower long-term [54]	Lower initial cost, higher follow-up [54]

#### 2.3.2. Covered Stents vs. Bare Metal Stents in Endovascular Treatment

Covered stents and bare metal stents differ in their application and risk profiles when used in endovascular management of aortic atresia or aortic coarctation—covered stents provide a protective barrier to the vessel wall, reducing the risk of acute aortic wall injury, dissection, rupture, and aneurysm formation, making them preferred in high-risk or complex lesions. In contrast, bare metal stents lack this protective layer, offering adequate dilation in lower-risk cases and growing patients, yet they carry a greater risk for post-procedural vascular complications such as aneurysm formation or dissection, especially in fibrotic or calcified segments [43, 55].

In clinical practice, covered stents are preferred for patients with fragile aortic walls, or conditions such as Turner syndrome, particularly in situations where acute aortic complications or surgical interventions pose significant risks. Conversely, bare metal stents are considered a safe and effective option for straightforward lesions in older children or adults when vessel integrity is robust and the risk of rupture is minimal. However, ongoing monitoring is essential to detect late complications, such as restenosis or aneurysm development. The use of covered stents may necessitate staged expansion or re-intervention to reduce pressure gradients and optimize outcomes. In contrast, bare stents may require modification and close follow-up if suboptimal expansion or wall injury occurs, with the potential for recoarctation if vessel anatomy is not fully addressed [56].

Branch vessel coverage is a contraindication for using a covered stent when the covered segment includes critical aortic branches such as the carotid, subclavian, vertebral, or mesenteric arteries, because occlusion of these vessels can lead to serious ischemic complications affecting cerebral, upper limb, or visceral perfusion. Specific contraindications include situations where the stent would obstruct the origin of the left subclavian artery or brachiocephalic trunk, or when vital side branches supply essential organs and collateral circulation is inadequate. In these cases, bare metal stents or advanced fenestrated and branched grafts are preferred to preserve flow to these vessels, and careful preprocedural imaging is necessary to assess individual risks and anatomical suitability for covered stent deployment [35].

#### 2.3.3. Surgical vs. Endovascular Treatment Effect on Post-Procedural Residual Hypertension

Studies show that surgical approaches like end-to-end anastomosis have lower rates of late hypertension, fewer requirements for ongoing antihypertensive medication, and greater aortic compliance, while stent implantation, although providing rapid anatomical relief, can reduce aortic elasticity, increase pulse wave impedance, and result in a higher need for hypertension therapy, especially in older patients or those with recurrent coarctation [57,58]. Early surgical repair, ideally before 1.5 years of age, is associated with lasting normotension and improved survival, but up to one-fifth of patients can still develop hypertension a decade or more post-surgery. In contrast, endovascular treatments offer immediate improvement in the majority of patients but up to one-third of adults may continue to require antihypertensive therapy—even when the anatomical result is optimal, stented segments may experience loss of compliance and impaired aortic pulsatility, contributing to persistent or late-onset hypertension [57]. While both surgical and endovascular techniques effectively treat aortic coarctation, surgical repair is associated with superior long-term blood pressure outcomes, and endovascular repair with faster recovery but greater risks for persistent hypertension, making individualized treatment selection critical for optimizing both anatomical and hemodynamic results and ensuring durable blood pressure control over time [59,60].

Hypertension remains a common late complication in both groups, but early surgical intervention leads to more patients remaining normotensive decades later, whereas one-third of adults after stenting can continue to experience hypertension despite anatomical success. The choice of technique should consider patient age, anatomy, and risk factors, but overall, surgical repair is linked to a more favorable long-term blood pressure profile after coarctation correction [59].

Surgical repair for aortic coarctation, particularly end-to-end anastomosis or patch procedures, generally results in lower recoarctation rates compared to endovascular treatments such as balloon angioplasty or stenting. The risk of reintervention is significantly lower after surgical correction, while late restenosis, somatic growth leading to stent mismatch, stent fracture, and aneurysm formation contribute to higher risk of recoarctation after endovascular procedures, especially in younger patients or those with long-segment or complex anatomy [57,61]. One-year reintervention rates are lower for surgery (98.7%) compared to endovascular repair (88.2%) and, over longer-term follow-up, recoarctation tends to occur more frequently following balloon angioplasty and in pediatric patients who may outgrow their implanted stents—stent implantation demonstrates better durability than balloon angioplasty but still falls short of surgical outcomes in terms of recoarctation, with most late events in adults relating to aneurysms and stent issues, while children are more likely to require staged reinterventions due to vessel growth [57].

Procedural factors contributing the most to higher recoarctation rates after stenting include young age at initial intervention, undersized or underexpanded stent, inadequate adaptation for future somatic growth (leading to size mismatch), use of first-generation or rigid stent designs like the PALMAZ stent, long stented aortic segments, suboptimal coverage of the lesion, high initial residual pressure gradient, irregular or gothic arch geometry, and occurrence of stent fractures or migration [61]. Children undergoing stenting before 12 years of age are at higher risk due to ongoing vessel growth that may necessitate repeated procedures for optimal sizing; stent underexpansion and insufficient diameter relative to future vessel size increase the likelihood of restenosis, while stenting irregular, severely narrowed, or tortuous anatomy raises the chance for poor apposition and late obstruction. Long lesions or those with unfavorable arch morphology require longer stents, which paradoxically increase the risk for restenosis and aortic complications such as aneurysm formation at proximal or distal edges. Early balloon dilation before stenting, high expansion pressures, and residual narrowing at implantation further compound the risk for reintervention [62].

Older, rigid stent designs such as the PALMAZ stent and early-generation bare metal stents most increase the risk of recoarctation after coarctation of the aorta (CoA) stenting—these stents are associated with a higher rate of restenosis, stent fracture, and aneurysm formation, especially when used in young patients who later require adaptation for somatic growth or in long lesions with severe narrowing. Bare metal stents, in contrast to covered stents, show higher rates of late restenosis, repeat intervention, and complications from insufficient aortic wall protection, while covered balloon-expandable stents (CBSs) demonstrate lower recoarctation and reintervention rates, with added benefit in high-risk or complex cases [63]. Stents with smaller initial balloon size, residual gradients > 10 mmHg after placement, use in complex or atretic lesions, and those placed in children < 15 years of age are linked to higher likelihood of recoarctation and need for future redilation or replacement. For optimal long-term outcomes, careful selection of stent type, size, and procedural technique is essential, with covered stents providing superior protection and lower incidence of recoarctation in most challenging anatomies [64].

## 3. Discussion

Aortic atresia is an extreme form of aortic coarctation with a very low prevalence in adults [6]. Adults with aortic atresia are usually asymptomatic due to extremely rich collateral circulation. The diagnosis of this pathology is generally made during extensive investigations for the diagnosis of hypertension [65].

There are currently no randomized controlled trials (RCTs) or large direct head-to-head comparative trials specifically focused on surgical versus endovascular treatment of aortic atresia in adults or children. Most evidence comes from retrospective cohort studies, case series, and systematic reviews, often with limited sample sizes due to the rarity and complexity of aortic atresia.

The treatment of these cases was, until recently, exclusively surgical. The first case was described in 1944 by Crafoord in Sweden [66]. Since then, various surgical approach techniques have been proposed [67]. In recent years, interventional repair techniques have been developed using balloon angioplasty or stenting. Surgery in newborns < 6 months, surgery or balloon angioplasty in children < 6 years, and stent implantation over 6 years are excellent alternatives to surgery alone. Balloon angioplasty has a high re-intervention rate of over 50% and should be used either as an emergency procedure or, in some cases, as a “bridge” to stenting [37,60]. In aortic atresia, especially when there is no permeable lumen, the most important part of the stenting procedure is the passage of a guidewire from the upper to the lower portion of the aorta. One study presented a cohort of 40 patients with aortic atresia, aged between 32 and 63, who were treated with covered stents. A radiofrequency system (Baylis MedComp Inc, Montreal, Canada), consisting of a Nykanen 0.024″ guidewire and coaxial microcatheter, penetrated the atretic segment [27]. Over an average follow-up period of 19 months, no complications were observed, and most patients no longer required antihypertensive treatment shortly after stenting. Another study described the penetration of the atretic segment using the transseptal Brockenbrough needle or the hard end of an angioplasty guidewire [65]. Both studies used covered stents after crossing the atretic segment and successive dilations. In a randomized clinical trial involving 120 patients with coarctation of the aorta, covered stents were not found to be superior to simple metallic stents. The latter had a higher recoarctation rate, but this did not reach statistical significance [68].

Covered stents were developed to address complications from simple balloon angioplasty or metallic stent implantation. They are preferred for patients with severely constricted coarctation or aortic atresia, those with aortic aneurysms, and elderly individuals with fragile aortic integrity [69].

The primary concern raised by using covered stents is the closure of collateral branches, the vast majority of which are asymptomatic; however, in some cases, hybrid strategies must be employed.

Aortic rupture or dissection complications are usually rare after interventional procedures. This is due to the recent advancements in interventional materials and techniques. Most complications relate to the vascular access site, but restenosis, aneurysm formation, stent fracture, and stent migration can also occur [37,70].

Balloon angioplasty was first reported in 1982 for coarctation treatment [71], but it can have a high rate of restenosis and aneurysm formation requiring further intervention [72], so it is rarely used today. Stent placement had a procedural success rate of 99% in the Coarctation of the Aorta Stent Trial (COAST), with no increased incidence of complications (6/105 patients developed aortic aneurysm and 2/105 patients stent fractures at one-year follow-up) [73].

After stent implantation, the residual gradient is crucial to patients’ long-term prognosis. It is anticipated that at the end of the procedure, the residual gradient will be less than 10 mmHg, with values above this correlating to an increased cardiovascular risk and persistence of hypertension [74].

While adapting CTO techniques to penetrate the atretic segment is potentially safer and more effective than using the transseptal puncture needle or the hard part of the angioplasty guidewire, a randomized trial is needed for confirmation.

Data from direct comparison of endovascular procedures as well as the selection of surgical versus interventional approaches present several limitations and conflicting results, particularly regarding the choice between covered and bare stents. Although covered stents have demonstrated a reduced risk of post-procedural aneurysm formation, they carry the potential drawback of occluding important collateral arteries, especially in cases with extensive collateralization, possibly compromising spinal or visceral perfusion. Therefore, the decision to employ covered stents should be tailored to the individual patient’s anatomy and collateral vasculature profile [39,75].

Notably, some studies have found no significant superiority of covered stents over bare-metal stents in terms of overall outcomes for aortic coarctation and atresia. For instance, a randomized trial involving 120 patients who received either a covered stent, or a bare metal stent revealed that while covered stents reduced aneurysm formation rates, bare-metal stents were associated with a higher—although not statistically significant—rate of recoarctation [68]. However, these studies often lack sufficient long-term data, and direct comparisons in the context of aortic atresia, as opposed to simple coarctation, remain sparse.

Similarly, while surgical approaches remain the gold standard in neonates and complex cases [76], the higher perioperative risk and invasiveness have justified wider adoption of endovascular strategies in adults [77]. The less invasive nature of endovascular repair translates to more desirable outcomes and a marked decrease in morbidity [78], without the need for extracorporeal circulation, profound hypothermia, and circulatory arrest [79].

The literature is limited by the rarity of true aortic atresia in adults and the heterogeneity of patient populations and technical approaches. Most published experiences are single-centre series or observational cohorts, which preclude the robust generalization of results and underpower the detection of rare complications, such as stent fracture or migration. Furthermore, head-to-head comparisons of surgery versus stenting in aortic atresia are lacking, and most evidence is extrapolated from broader coarctation cohorts.

Overall, while stent implantation—particularly with covered stents—offers an attractive, less invasive alternative with encouraging mid-term outcomes, further multicentre studies with longer follow-up and standardized endpoints are needed. Particular attention should be paid to long-term risks of restenosis, stent-related complications, and the impact on collateral circulation. Selection between covered and bare stents, as well as between surgical and interventional repair, should be individualized, guided by careful anatomical and clinical assessment, and consideration of the limitations and gaps in the current evidence base.

Comparisons between surgery and endovascular repair in aortic atresia and near-atresia are mostly limited to observational cohorts or case reports, sometimes presenting pooled institutional experiences. Treatment decisions are individualized based on anatomy, institutional expertise, and comorbidities, informed by observational data and not by direct RCT-level evidence.

Evidence-based medicine (EBM) in aortic atresia treatment involves integrating the best available clinical data, expert consensus, and patient characteristics to decide between surgery and endovascular options. Due to the absence of randomized trials, recommendations rely heavily on observational evidence, systematic reviews, and expert guidelines [34].

We need to individualize the decision based on age, comorbidities, anatomy, risk factors, and institutional experience. Shared decision-making with the patient and caregivers must be considered, discussing potential benefits (durability, less reintervention) and risks (invasiveness, recovery time) of each approach.

### 3.1. Gaps in Knowledge and Future Directions

Gaps in knowledge regarding surgical treatment for aortic atresia and coarctation center around understanding the best timing and most appropriate technique for varying anatomical presentations, quantifying long-term risks like recoarctation, aneurysm formation, and persistent hypertension, and determining which patients benefit most from extensive versus conservative repair strategies, while outcomes can be affected by patient age, lesion complexity, and associated defects and lack of validated risk models or standardized treatment algorithms means that practice is often variable—many existing studies have limited follow-up duration and small, single-center populations, making it difficult to generalize results and predict late complications or reintervention needs [80].

Significant gaps in knowledge persist in the contemporary management of aortic atresia, particularly regarding prenatal intervention outcomes, optimal surgical approaches, long-term patient prognosis, and criteria for advanced therapies. Evidence on which fetal populations derive the most significant benefit from prenatal intervention remains limited, and there is insufficient risk stratification to guide clinical decision-making. Moreover, the long-term outcomes following established surgical procedures—most notably the Norwood operation—have reached a plateau, highlighting the need for robust risk assessment models and longitudinal studies that span beyond infancy, especially for those with complex anatomical features [81].

Current controversies surround the timing and selection of surgical techniques, such as the choice between the modified Blalock-Taussig shunt and right ventricle-pulmonary artery shunt, as interstage mortality remains a concern and surgeon experience influences outcomes. Alternative strategies like hybrid procedures also require further comparative evaluation in high-risk neonatal populations. Additionally, there is a lack of consensus regarding candidacy for heart transplantation and mechanical circulatory support devices in pediatric patients with complex cardiac anatomy [82].

In the landscape of endovascular management for aortic atresia and coarctation, challenges persist with the absence of robust, long-term evidence supporting the durability and safety of stent grafts and angioplasty, especially for pediatric and anatomically complex patients, where risks of recoarctation, aneurysm development, hypertension, and device migration remain concerns. Patient selection remains a moving target, as clear criteria for recommending endovascular over surgical or hybrid repair are under ongoing investigation; complexities in anatomy or the recurrence of coarctation further complicate decision-making. The lack of randomized trials directly comparing outcomes between surgical and endovascular approaches hinders consensus on best practices and leaves the impact on cardiovascular health, survival, and psychosocial function inadequately characterized in the endovascular cohort [20,83,84].

Technical limitations continue to influence outcomes because endovascular techniques must overcome issues related to vascular access, device suitability for diverse presentations, and risks to adjacent vessels; managing complications such as neurological events or the need for repeat intervention requires more standardized outcomes and vigilant follow-up. The next frontier involves the design and refinement of branched and fenestrated stent platforms, expanding the feasibility for complex arch and isthmus repair. At the same time, individualized hybrid approaches guided by multidisciplinary teams promise tailored therapy with improved anatomical and functional results [85].

Long-term multicenter studies with meaningful patient-centered endpoints are needed to address questions of recoarctation, cardiovascular complications, functional recovery, and quality of life; the integration of advanced imaging, robust risk stratification, and comprehensive registries may empower clinicians to develop dynamic care algorithms for selecting and timing interventions. Technical innovations will likely prioritize reducing procedural risk, optimizing device delivery, and ensuring that repairs support sustained vessel health and growth in all age groups, moving the field toward safer and more effective endovascular solutions [86].

Looking forward, future directions are focused on several promising avenues: the refinement of fetal cardiac interventions to maximize potential for biventricular repair, the development and application of regenerative medicine strategies—such as stem cell therapy to support myocardial function—and the advancement of pediatric mechanical circulatory support technologies, including ventricular assist devices as bridges to transplantation or palliation. There is also an increased emphasis on personalized risk stratification methods that account for patient-specific and socioeconomic variables, coupled with a growing body of patient-centered research aimed at improving quality of life and informing clinical guidelines. Innovations in less invasive hybrid procedures, combining surgical and catheter-based modalities, continue to be explored, with multicenter trials necessary to establish efficacy and optimal indications [87].

Emerging directions in the management of aortic atresia and aortic coarctation are rapidly shaped by innovation in diagnosis, device technology, and individualized therapy, as well as multidisciplinary care and rigorous outcome research—advancements in prenatal imaging and fetal cardiac intervention create opportunities for correcting obstructions before birth with procedures such as fetal aortic valvuloplasty, while bioengineered tissues and stem cell therapy raise the prospect of myocardial regeneration and long-term improved function. In pediatric and adult cohorts, the promise of biodegradable, growth-compatible, and dilatable stents offers solutions for aortic coarctation lesions that account for vessel growth and minimize late complications, with clinical trials actively exploring feasibility, safety, and benefit—new generations of stent grafts with branched and fenestrated designs also extend the reach of endovascular therapy to more anatomically complex aortic segments, aiming to reduce procedural risk and broaden eligibility [88].

Personalized, multidisciplinary decision-making leverages advanced cross-sectional imaging, patient-specific 3D modeling, and evolving clinical algorithms to guide selections among open, hybrid, or percutaneous approaches according to age, anatomy, and comorbidities—hybrid repairs that combine minimally invasive endovascular techniques with tailored surgical correction are notably gaining traction where complex pathology or recurrent disease challenges conventional management. Lifelong follow-up is essential for both congenital aortic atresia and coarctation patients, with careful monitoring of hypertension, neurodevelopmental outcomes, and cardiovascular risk. The development of multicenter, longitudinal registries and patient-reported outcome measures is central for benchmarking new therapies and tracking the real-world impact of interventions across the lifespan [25,89].

The future of aortic atresia and coarctation management, therefore, lies in continuing to refine device technology, advancing minimally invasive strategies, individualizing care pathways, and sustaining research that illuminates long-term efficacy and quality of life for all patients.

### 3.2. Limitations of This Article

The article constitutes a narrative review rather than a systematic review, indicating that it does not comprehensively search for or synthesize all available evidence. This approach may introduce selection bias, as the selected studies were selectively included to align with our perspective while excluding contradictory evidence. The central focus of the article is a single case report with short-term follow-up, as opposed to findings derived from a large patient cohort or randomized trial. Consequently, the applicability and generalizability of the outcomes are constrained. The article underscores the rarity of aortic atresia in adults; however, the descriptions and recommendations are based on heterogeneous cases (in terms of age, anatomy, and comorbidities), which limit the ability to draw precise, universally applicable conclusions.

Widespread clinical adoption of the recommendations regarding aortic atresia diagnosis, treatment and follow-up would require further research, including controlled studies and longer-term outcome data.

## 4. Conclusions

In conclusion, thoracic aortic atresia in adults is a rare and complex condition for which treatment strategies have evolved from exclusively surgical approaches to include endovascular interventions inspired by chronic coronary occlusion techniques. While both modalities have unique advantages and limitations, no randomized trials or direct comparative studies are available to guide optimal management, resulting in reliance on case series, observational cohorts, and extrapolation from related conditions (aortic coarctation). Surgical repair remains the standard for neonates, infants, and patients with complex or extensive anatomical defects, owing to its durability and favorable long-term outcomes for blood pressure control and lower reintervention rates. Endovascular techniques—particularly percutaneous recanalization and stenting—are increasingly feasible in adults with discrete lesions, providing less invasive options and faster recovery, although with higher risks of persistent hypertension and reintervention, as well as technical limitations tied to anatomy and access. Current literature is constrained by small, heterogeneous patient populations and lacks robust comparisons between surgical and endovascular interventions for thoracic aortic atresia. Individualized treatment decisions are essential, factoring in patient age, anatomical complexity, comorbidities, and institutional expertise, with shared decision-making involving the patient and the medical staff. There is a need for multicenter, long-term studies focusing on durability, complication rates, and impact on collateral circulation. Advancements in device technology—such as branched and fenestrated stent platforms—and ongoing innovations in hybrid procedures hold promise for expanding treatment possibilities. Lifelong follow-up and the development of comprehensive registries remain key priorities for improving outcomes and benchmarking therapies. Overall, the management of adult thoracic aortic atresia should be personalized, integrating current evidence, multidisciplinary expertise, and evolving technical options to maximize safety, efficacy, and patient-centered outcomes while bridging enduring gaps in research.

## Data Availability

The original contributions presented in this study are included in the article. Further inquiries can be directed to the corresponding author.

## Abbreviations

The following abbreviations are used in this manuscript:

AA	Aortic Atresia
AV	Aortic Valve
BIB	Balloon-in-Balloon (catheter)
BP	Blood Pressure
BMW	Balance Middle Weight (guidewire, Abbott Vascular)
CT	Computed Tomography
CTO	Chronic Total Occlusion
ECG	Electrocardiogram
EF	Ejection Fraction
F	French (catheter size)
JR	Judkins Right (catheter)
LV	Left Ventricle
LVH	Left Ventricular Hypertrophy
MPA2	Multipurpose A2 (catheter)
MV	Mitral Valve
TTE	Transthoracic Echocardiogram
Confianza Pro 12	Asahi Intecc coronary guidewire
FineCross	Terumo microcatheter
Cheatham Platinum D’Vill	Covered stent (NuMed Inc.) Introducer sheath system (NuMed Inc.)
CT angiography	Computed tomography angiography
